# Arabidopsis Transporter ABCG37/PDR9 contributes primarily highly oxygenated Coumarins to Root Exudation

**DOI:** 10.1038/s41598-017-03250-6

**Published:** 2017-06-16

**Authors:** Jörg Ziegler, Stephan Schmidt, Nadine Strehmel, Dierk Scheel, Steffen Abel

**Affiliations:** 1 Department of Molecular Signal Processing, Leibniz Institute of Plant Biochemistry, D-06120 Halle (Saale), Germany; 2 Department of Stress and Developmental Biology, Leibniz Institute of Plant Biochemistry, D-06120 Halle (Saale), Germany; 3 Institute of Biochemistry and Biotechnology, Martin Luther University Halle Wittenberg, D-06120 Halle (Saale), Germany; 4 Department of Plant Sciences, University of California-Davis, Davis, CA 95616 USA

## Abstract

The chemical composition of root exudates strongly impacts the interactions of plants with microorganisms in the rhizosphere and the efficiency of nutrient acquisition. Exudation of metabolites is in part mediated by ATP-binding cassette (ABC) transporters. In order to assess the contribution of individual ABC transporters to root exudation, we performed an LC-MS based non-targeted metabolite profiling of semi-polar metabolites accumulating in root exudates of *Arabidopsis thaliana* plants and mutants deficient in the expression of ABCG36 (PDR8/PEN3), ABCG37 (PDR9) or both transporters. Comparison of the metabolite profiles indicated distinct roles for each ABC transporter in root exudation. Thymidine exudation could be attributed to ABCG36 function, whereas coumarin exudation was strongly reduced only in ABCG37 deficient plants. However, coumarin exudation was compromised in *abcg37* mutants only with respect to certain metabolites of this substance class. The specificity of ABCG37 for individual coumarins was further verified by a targeted LC-MS based coumarin profiling method. The response to iron deficiency, which is known to strongly induce coumarin exudation, was also investigated. In either treatment, the distribution of individual coumarins between roots and exudates in the investigated genotypes suggested the involvement of ABCG37 in the exudation specifically of highly oxygenated rather than monohydroxylated coumarins.

## Introduction

Root exudation is essential for plant growth and performance as the excreted compounds play critical roles in the establishment of mutualistic interactions with microorganisms, in pathogen defense, or in nutrient acquisition^1^. Among other transport systems, ATP-binding cassette (ABC)-type transporters often efflux these compounds into the rhizosphere^2–7^. In *Arabidopsis thaliana*, the ABC transporter family consists of 130 members, which are assorted into eight groups (A-H) according to overall size and arrangement of transmembrane domains (TMD) and nucleotide binding domains (NBD)^8, 9^. Members of subfamilies B, C, and G, which are also known as multidrug resistance (MDR), multidrug resistance associated proteins (MRP), and pleiotropic drug resistance (PDR) proteins, respectively, are full-size ABC transporters possessing each two TMD and two NBD. Specific functions have mainly been assigned to these transporters based on phenotypic and metabolic changes in the respective mutants. For example, ABC transporters of subfamily B are involved in auxin transport and distribution^10^, and members of group C have been implicated in the regulation of stomatal aperture and guard cell ion flux, as well as in the transport of glutathione conjugates^11–15^. However, some of these members also transport chlorophyll catabolites^12, 13^ and phytochelatins^16–18^ or are responsible for phytate accumulation^19^, which indicates a certain level of substrate promiscuity.

**Figure 1:**
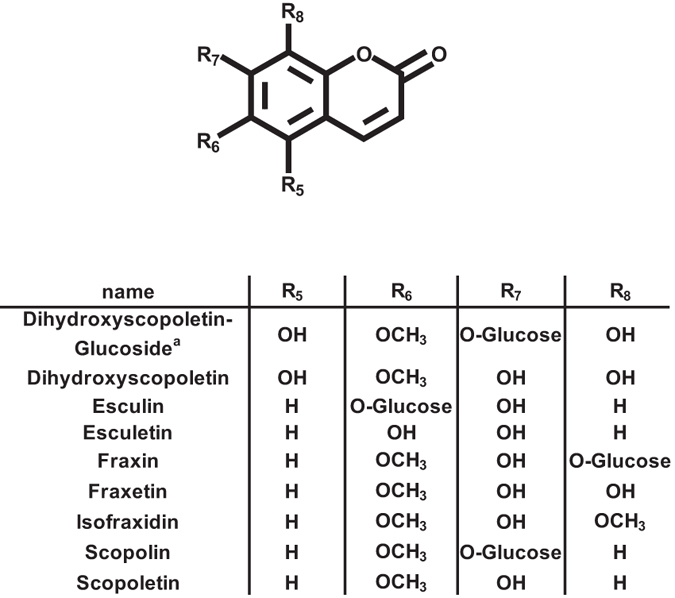
Chemical structures of coumarins measured in this study. ^a^Note that the positions of the substitutions in dihydroxyscopoletin and dihydroxyscopoletin glucoside have not been confirmed yet by NMR studies.

Functional promiscuity has also been described for ABCG36/PDR8/PEN3, a transporter involved in resistance of Arabidopsis to nonadapted and/or host-adapted pathogens^20–24^. It also acts as a cadmium extrusion pump in roots, thereby conferring resistance to heavy metal stress^25^. Furthermore, ABCG36 mediates efflux of the auxin precursor indole 3-butyric acid (IBA) from roots, as evidenced by hypersensitive root growth phenotypes of *abcg36* mutants in the presence of IBA or precursors of synthetic auxin analogues^26, 27^. Despite its multiple roles for root physiology, it has not yet been investigated whether ABCG36 contributes to root exudation. A function as a transporter of IBA and various auxinic compounds has also been assigned to ABCG37/PDR9, as evidenced by altered responsiveness of *abcg37* mutant plants to synthetic auxins and inhibitors of auxin transport, but not to indole-3-acetic acid (IAA), the endogenous auxin^27–29^. Interestingly, *abcg37* knockout lines also displayed hypersensitivity to iron (Fe) deficiency, which has been attributed to impaired coumarin excretion, especially of scopoletin^7^. Coumarins are a group of phenylpropanoid derived 1,2 benzopyrones exhibiting various patterns of methoxy and hydroxyl functions (Fig. 1) and predominantly occur as glucosylated forms in root tissue, whereas the respective aglycones are mainly detectable in root exudates. It has been reported that increased coumarin biosynthesis and exudation promotes plant Fe acquisition by chelating Fe^3+^ from insoluble Fe complexes, particularly in calcareous growth substrates^7, 30–36^. As such, *abcg37* mutants and *feruloyl-CoA 6′ hydroxylase1* (*f6′h1*) knockout lines that are incapable of coumarin biosynthesis show more severe Fe deficiency symptoms and accumulate less Fe when compared to wild type plants under Fe limiting conditions^7, 33, 34^. Decreased abundance of all analyzed coumarins in root exudates of *abcg37* mutants suggested broad substrate specificity of ABCG37 with respect to the substitution pattern of coumarins^7^. Similarily, exudates of *f6′h1* knockout lines do not contain any coumarins as they are unable to synthesize coumarins^33^. As such, both studies did not provide hints about specificities towards individual coumarins with respect to exudation.

Recently, we reported that coumarin profiles are also profoundly altered in root exudates of phosphate (Pi) deprived plants^37^. We observed higher accumulation of a subset of coumarin derivatives, whereas the abundance of others, mainly highly oxygenated coumarins, was decreased when compared to exudates of Pi replete roots. This is in contrast to Fe deficient growth conditions, which promote accumulation of all coumarins, foremost of highly oxygenated coumarins or methylated derivatives there of ^7, 33, 34^. The different coumarin profiles indicate differential regulation of biosynthesis and/or exudation of individual coumarins, which might also be reflected by the existence of transport systems with selectivity for coumarin compounds.

In this study, we compared root exudation of wild-type (Col-0) plants, of *abcg36* and *abcg37* knockout lines as well as of the *abcg36;abcg37* double mutant by liquid chromatography mass spectrometry (LC-MS)-based metabolite profiling of semi-polar metabolites. Non-targeted metabolite profiling indicated that both ABC transporters mediate the exudation of specific as well as common metabolites based on the absence of *m/z*-retention time pairs (features) in root exudates of the mutants compared to the wild-type. The unexpected observation of similar scopoletin levels in root exudates of *abcg37* and wild-type seedlings prompted us to explore in more detail coumarin profiles in root exudates by a targeted LC-MS approach. Our experiments suggest selectivity of ABCG37 for the exudation of individual, mainly highly oxygenated coumarins.

## Results

### Comparative Non-Targeted Profiling of Semi-Polar Metabolites in Root Exudates

To investigate the contribution of ABCG36/PDR8 and ABCG37/PDR9 transporters to root exudation, we cultivated wild-type (Col-0) seedlings, *pdr8-1* and *pdr9-2* knockout lines, as well as the *pdr8-1;pdr9-2* double mutant in the 96-well hydroponic system as described previously^37^. After germination and 4 days of growth, seedlings were transferred to fresh media and exudates were collected after additional 7 days. At the time of harvest (day 11), plant growth parameters such as root length and fresh weight of the entire seedlings, shoots, and roots were indistinguishable between the wild-type and single mutants, whereas the *pdr8-1;pdr9-2* double mutant exhibited reduced seedling (75%, *P* = 0.02), shoot (71%, *P* = 0.03) and root (65%, *P* = 0.03) fresh weights as well as shorter primary root length (77%, *P* = 0.0004) when compared to the wild-type (Supplementary Fig. S1). The germination rate of *pdr8-1;pdr9-2* seeds was about half compared to the other genotypes.

**Figure 2:**
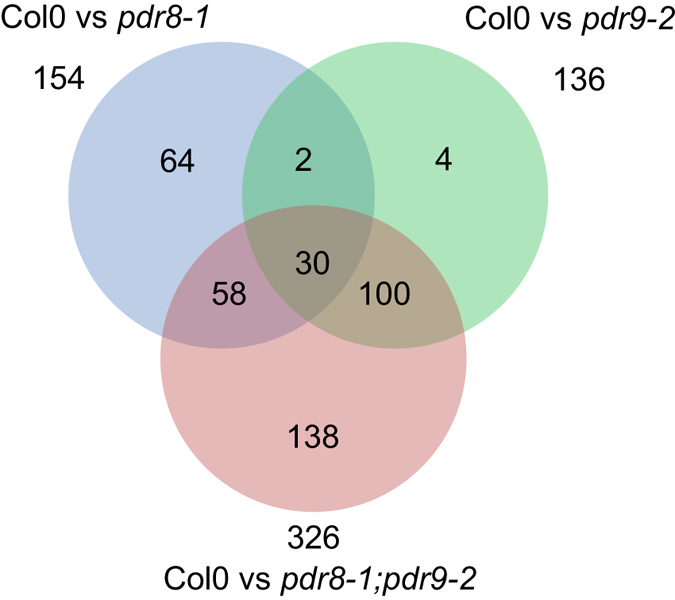
Venn diagram displaying the number of differential features in root exudates between the wild-type vs. mutant comparisons, and the number of overlapping features.

For root exudate analysis, we collected from 3 independent experimental setups 13 samples each for wild-type and *pdr9-2*, 15 samples for *pdr8-1*, and 8 samples for *pdr8-1;9-2* (each sample comprised the media pooled from 8 single seedlings). For controls, we included 15 samples consisting of the media collected from blank wells. Two samples of *pdr8-1;pdr9-2* had to be omitted from subsequent analysis because of low data quality (retention time shift > 5 s and intensity deviation > 30% of the internal standard, 2,4 dichlorophenoxy acetic acid), resulting in total of 124 datasets (all replicate samples, positive and negative ionization, see Supplementary Table S1). After data processing, we obtained 1,452 features (*m/z*-retention time pairs), which comprised 854 and 598 features for positive and negative ionization modes, respectively (Supplementary Data S1). We detected 154 features with differences in abundance (>1.5 fold difference, *P* < 0.01) between wild-type and *pdr8-1*, 136 features between wild-type and *pdr9-2*, and 326 features between wild-type and *pdr8-1;pdr9-2* (Fig. 2). Compared to wild-type, 16, 1, and 7 features were of higher abundance in *pdr8-1*, *pdr9-2* and the double mutant, respectively. The feature with higher intensity in *pdr9-2* exudates was absent in wild-type exudates (i.e. <1.5 fold, *P* > 0.01 compared to blank samples). Three differential features of the wild-type vs. *pdr8-1* comparison were not detectable in root exudates of *pdr8-1*. This was the case for 16 and two features in the double mutant and *pdr9-2*, respectively. Almost all differential features between wild-type and *pdr9-2* (130/136, 96%) were found among the differential signals between wild-type and the double mutant, but only 55% (88/154) of the wild-type vs. *pdr8-1* comparison (Fig. 2). Interestingly, the comparison between wild-type and the double mutants yielded decidedly more differential features (138) than the sum of the differential features in the comparison with the single mutants (70). As such, the abundance of 138 features was different only between root exudates of wild-type and *pdr8-1;pdr9-2*. Both single mutants shared 32 differential features when compared to wild-type, of which almost all (30) were contained in the wild-type vs. double mutant comparison (Fig. 2).

### Identification of Features Specific for ABCG36 and ABCG37

To identify features that are related to ABCG36/PDR8 transport substrates, we first determined the number of differential features between wild-type and *pdr8-1* exudates. These features should also be among the differential features between wild-type and the double mutant as well as between *pdr8-1* and *pdr9-2*. Conversely, the remaining features should not exhibit differential abundance between root exudates of wild-type and *pdr9-2* or between *pdr8-1* and the double mutant. This filtering resulted in 27 *pdr8*-specific features, 22 of which were more abundant in wild-type root exudates. Applying the same procedure to the respective *pdr9-2* comparisons yielded 64 *pdr9*-pecific features, which were all more abundant in root exudates of wild-type plants and may thus be derived from ABCG37/PDR9 substrates (Supplementary Data S1).

**Figure 3:**
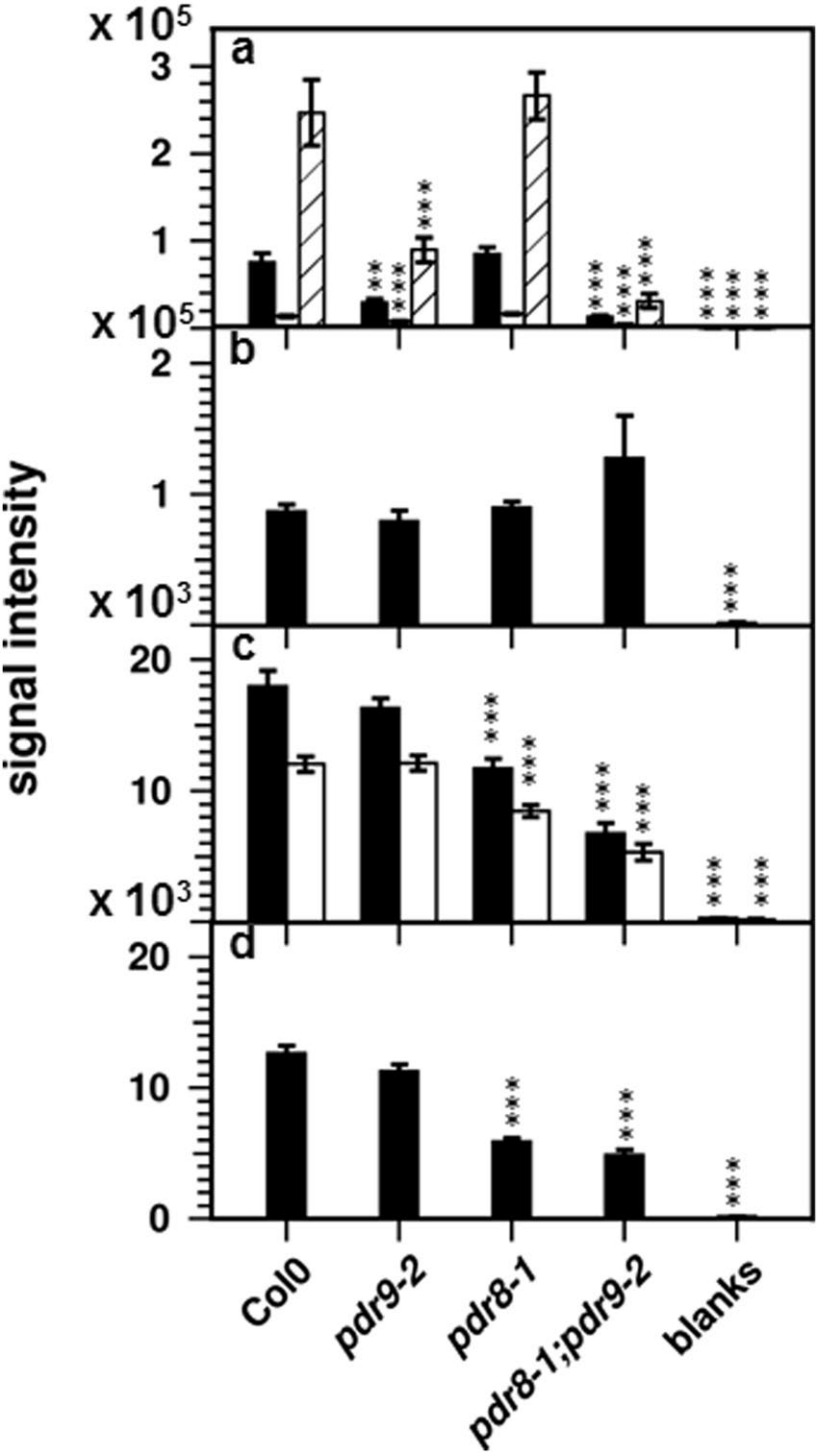
Signal intensities of selected features. (**a**) dihydroxyscopoletin (*m/z* 210.014 at 199 s [black bars], *m/z* 374.963 at 199 s [white bars], and *m/z* 225.039 at 199 s [hatched bars]); (**b**) scopoletin (*m/z* 193.049 at 265 s); (**c**) scopolin (*m/z* 377.084 at 193 s [black bars] and *m/z* 399.089 at 193 s [white bars]); (**d**) thymidine (*m/z* 241.082 at 74 s). Error bars denote SEM (*n* ≥ 6). Significance analysis between mutants and Col0 was performed by Student’s *t*-test (two tailed, equal variance): *P ≤ 0.05; **P ≤ 0.01; ***P ≤ 0.001.

In order to obtain hints about the identity of the specific features related to ABCG36/PDR8 and ABCG37/PDR9 transport substrates, we compared those with the more than 100 characterized or identified features occurring in Arabidopsis root exudates^38^. In the entire dataset, several, previously annotated features could be detected, among them features annotated as amino acids, indolic compounds, oligolignols, phenylpropanoids and fatty acid derivatives (highlighted in green in Supplementary Data S1). However, none of the ABCG37-related features and only one feature related to ABCG36 (thymidine *m/z* 241.0821 at 74 s) are present in this list. Interestingly, we did not detect scopoletin or derivatives thereof among the 64 ABCG37-related features although scopoletin was reported to be a substrate of ABCG37^7^ and several scopoletin derivatives are listed by Strehmel *et al*.^38^. To exclude the possibility that the absence of scopoletin as an ABCG37-related signal was due to the applied filtering procedure, we analyzed the raw data for signal intensities of the scopoletin feature ([M + H]^+^ *m/z* 193.0491 at 265 s). As shown in Fig. 3b, the scopoletin signal was clearly detectable in root exudates of wild-type plants, however, its intensity was similar for all such samples. There were also no differences for the scopolin signals ([M + H + Na]^+^ *m/z* 377.084 at 193 s; [M−H + formic acid]^−^ *m/z* 399.0893 at 193 s) between wild-type and *pdr9-2* exudates (Fig. 3c). The data rather indicate a decrease of scopolin in root exudates of *pdr8-1* plants. Since the scopolin signals were different in the comparison of *pdr8-1* and the double mutant they were not listed as *pdr8*-specific features. However, we noticed the presence of two features at 199 s ([M−H + NaFA + KFA]^*−*^ *m/z* 374. 963 and [M + H]^+^ *m/z* 210.015) in the list of the *pdr9-2* specific signals, which we previously annotated as the sodium formate and potassium formate adduct of the coumarin dihydroxyscopoletin and as dihydroxyscopoletin after loss of a ^•^CH3 radical^37^. As shown in Fig. 3a, the intensities of these signal were clearly lower in root exudates of *pdr9-2* (*P* < 0.01) and the double mutant (*P* < 0.0006) compared to wild-type, whereas no change could be observed for root exudates of *pdr8-1*.

**Figure 4:**
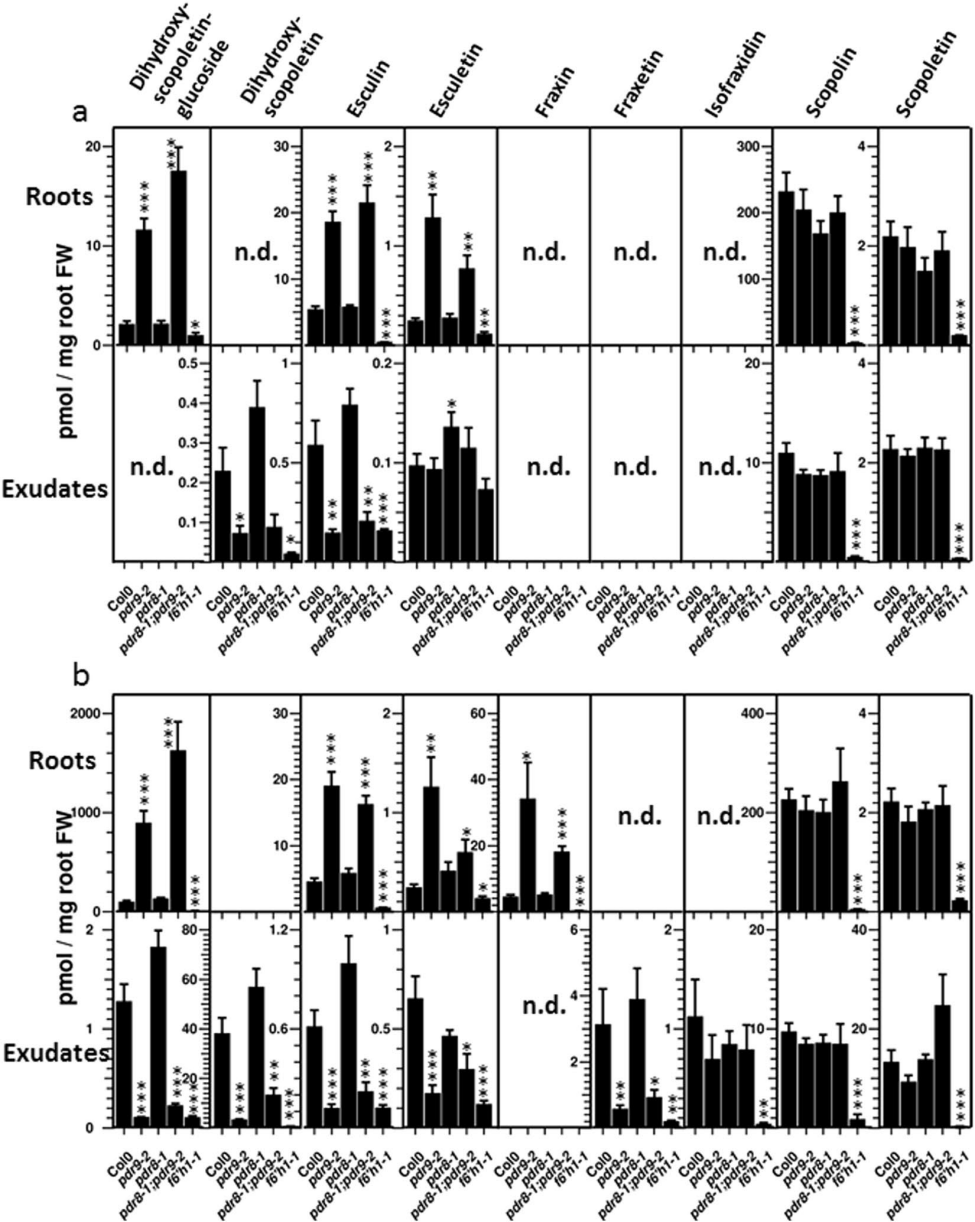
Coumarin levels in roots (upper panels) and root exudates (lower panels) of seedlings cultivated under Fe sufficient (**a**) and Fe deficient (**b**) conditions. Error bars denote SEM (*n* = 8). Significance analysis between mutants and the wild-type was performed by Student’s *t*-test (two tailed, equal variance): *P ≤ 0.05; **P ≤ 0.01; ***P ≤ 0.001. n.d.: not detectable.

In order to further confirm the identity of the tentative dihydroxyscopoletin, we compared its MS spectra with those of fraxetin and scopoletin (Supplementary Fig. S2). Collision induced dissociation in the positive ionization mode yielded mainly neutral losses of CO, CH_3_OH, as well as **·**CH_3_ radical elimination, generating fragments of *m/z* of 178, 150, 133, and 122 from the [M + H]^+^ ion of scopoletin (*m/z* 193). Fragmentation of fraxetin (*m/z* 209 [M + H]^+^) yielded an analogous series of fragments exhibiting an increase by 16 mass units (*m/z* 194, 166, 149, 138). The mass spectrum of the tentative dihydroxyscopoletin (*m/z* 225 [M + H]^+^) showed several fragments with an increased mass by 16 mass units compared to fraxetin, and by 32 mass units compared to scopoletin (*m/z* 210, 182, 165, 154). The strong similarity of the mass spectra with a shift in fragment sizes of 16 and 32 mass units compared to authenticated fraxetin and scopoletin, as well as the strongly increased intensities of the tentative dihydroxyscopoletin signal after Fe deficiency, and its absence in the coumarin deficient *f6′h1-1* mutant (Fig. 4), strongly suggests its identity as dihydroxyscopoletin. However, the parent ion of *m/z* 225 ([M + H]^+^) was not present among the *pdr9-2* specific features, although the signal intensities of this ion were also strongly decreased in exudates of *pdr9-2* and double mutant plants, but unchanged in exudates of *pdr8-1* plants (Fig. 3a). Since the difference in signal intensities between *pdr9-2* and double mutants plants was more than 1.5 fold, the parent ion of *m/z* 225 escaped our filtering procedure.

We did not detect differences in signal intensities between the genotypes for two features which were previously annotated as coumarinolignans^38^. Despite similar masses (*m/z* 387.1075 [M + H]^+^ and *m/z* 385.0937 [M-H]^−^) and the identical empirical formular (C_20_H_18_O_8_), the mass spectra showed that these are not cleomiscosin A and B, which were recently identified in Arabidopsis root exudates^36^, but rather cross coupling products of syringyl alcohol and esculetin.

### Comparative Targeted Profiling of Coumarins in Roots and Root Exudates

**Figure 5:**
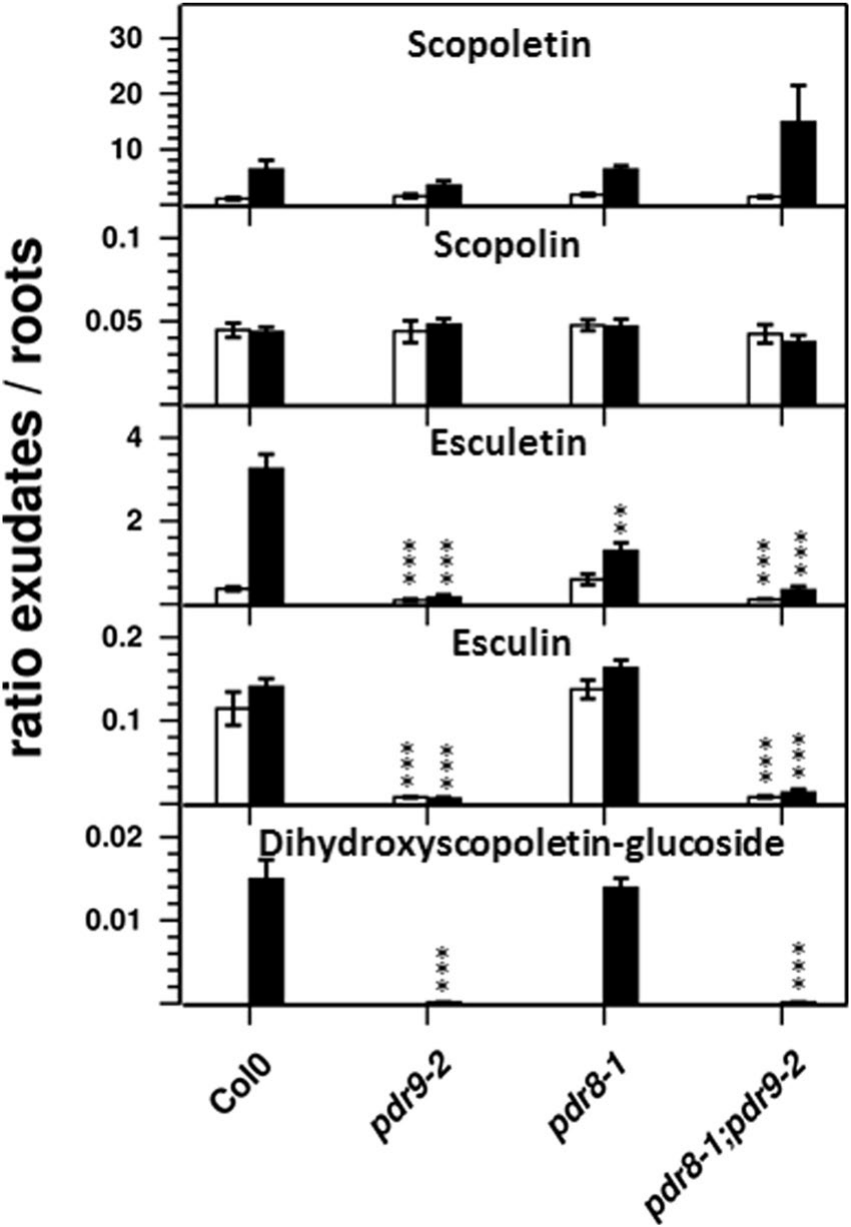
Ratios between coumarin levels in root exudates and roots of Fe sufficient (open bars) and Fe deficient (solid bars) plants. Error bars denote SEM (*n* = 8). Significance analysis between mutants and the wild-type was performed by Student’s *t*-test (two tailed, equal variance): *P ≤ 0.05; **P ≤ 0.01; ***P ≤ 0.001

Because our non-targeted metabolite profiling approach did not detect all coumarin compounds in root exudates that were reported as ABCG37/PDR9 transport substrates^7^, we used a more sensitive targeted profiling approach to analyze coumarin levels in root exudates and root extracts. Additionally, we subjected the seedlings to Fe deficiency, which increases the synthesis and exudation of most coumarins in wild-type plants^7, 33, 34^. We included the coumarin-deficient *f6′h1-1* mutant in this study in order to discriminate coumarin specific signals from signals derived from unrelated substances. With the exception of esculetin in root exudates (Fig. 4a), all coumarin signals are significantly lower in *f6′h1-1* plants compared to wild type, confirming the specificity of the targeted analysis. Scopolin exhibits the highest level of all coumarins, predominantly in roots, whereas its aglycone, scopoletin, is evenly distributed between roots and root exudates (Figs 4a and 5). Similar to scopolin, esculin was mainly present in roots and only a small fraction of the esculin pool was detected in root exudates. Esculetin, the aglycone, was barely detectable in wild-type. Dihydroxyscopoletin was exclusively detected in root exudates, whereas the occurrence of its glucoside was restricted to roots (Fig. 4a). Consistent with the result of the non-targeted profiling approach, scopoletin levels in root exudates of the wild-type and the three ABC transporter mutants, *pdr8-1*, *pdr9-2*, and *pdr8-1;pdr9-2*,were similar, as was its glucoside, scopolin. Likewise, we did not observe significant changes for both compounds in roots. Esculin levels were reduced in root exudates of *pdr9-2* and of the double mutant, but increased in roots. Esculetin exhibited a similar pattern in roots, but in exudates a change could only be detected for *pdr8-1*. Prominent changes were observed for dihydroxyscopoletin and its glucoside. In root exudates, the level of dihydroxyscopoletin was reduced to 34% in *pdr9-2*. The decreased (55%) and increased levels (1.8 fold) in root exudates of the double mutant and *pdr8-1* line, respectively, were not highly statistically significant (*P* = 0.08; *P* = 0.07). The decrease of the aglycone in root exudates of *pdr9-2* and *pdr8-1;pdr9-2* was mirrored by an increase of the glucoside in the respective roots; it strongly accumulated in roots of *pdr9-2* (6-fold) and *pdr8-1;pdr9-2* (9-fold) plants.

### Comparative Targeted Coumarin Profiling under Fe Deficiency

Coumarins profoundly accumulate in root exudates during Fe deficiency, and the contribution of ABCG37 to coumarin exudation has mainly been established after plants were subjected to Fe limiting conditions^7, 33, 34^. After exposure of wild-type roots to Fe-free media for 7 days, dihydroxyscopoletin content of exudates excessively increased to levels that were almost 200-fold higher compared to +Fe control conditions, whereas esculetin and scopoletin accumulated 6-fold and 5-fold, respectively (Fig. 4a and b). Additionally, fraxetin and isofraxidin, which escaped detection under control conditions, accumulated to appreciable levels. In roots, dihydroxyscopoletin glycoside accumulation was almost as strong as the increase of the aglycone in exudates. We were also able to detect the glucoside in root exudates, although at a low level compared to the endogenous root content (1%). As with fraxetin in root exudates, its glucoside, fraxin, was only detectable in roots under Fe limiting conditions. The alterations in coumarin profiles between the transporter mutants and the wild-type under Fe deficiency were similar to the changes observed under control conditions (Fig. 4b). Dihydroxyscopoletin glucoside and esculin contents were strongly increased in roots of *pdr9-2* and *pdr8-1;pdr9-2* plants whereas dihydroxyscopoletin, its glucoside, and esculin levels were strongly decreased in root exudates. After Fe deficiency, it was possible to detect a decrease in abundance of esculetin in root exudates of *pdr9-2* plants and of the double mutant. Furthermore we also observed increased levels of fraxin in roots but decreased levels of fraxetin in exudates of these plants. Again, no changes in the levels of scopolin and scopoletin in roots and root exudates could be observed for the transporter mutants. The decreased levels of isofraxidin in root exudates of the transporter mutants displayed low statistical significance (*P* > 0.17).

**Figure 6:**
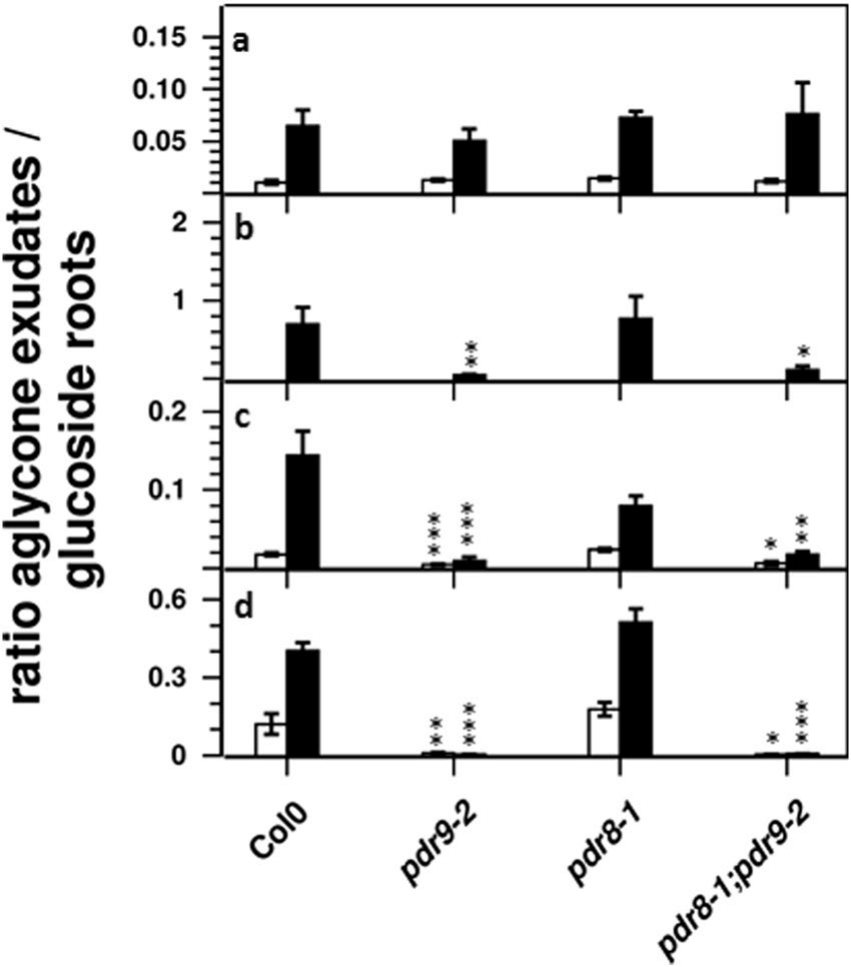
Ratio between levels of aglycones in exudates and glucosides in roots of Fe sufficient (open bars) and Fe deficient (solid bars) plants. (**a**) Scopoletin/Scopolin; (**b**) Fraxetin/Fraxin; (**c**) Esculetin/Esculin; (**d**) Dihydroxyscopoletin/Dihydroxyscopoletin-Glucoside. Error bars denote SEM (*n* = 8). Significance analysis between mutants and Col0 was performed by Student’s *t*-test (two tailed, equal variance): *P ≤ 0.05; **P ≤ 0.01; ***P ≤ 0.001.

Based on these data we calculated the distribution of each coumarin compound between exudates and roots (Fig. 5). As expected, the glucosides esculin, scopolin, and dihydroxyscopoletin-glucoside were more abundant in roots. Interestingly, the distribution of the aglycone esculetin between exudates and roots was similar to the distribution of its glycone esculin under Fe sufficient conditions irrespective of the genotype. In contrast, the aglycone scopoletin was detected at slightly higher levels in exudates of Fe sufficient seedlings. Upon Fe deficiency, the fraction of scopoletin and esculetin in exudates strongly increased, whereas the distribution of the respective glucoside remained unaltered. The ratios between agylcones in root exudates and the corresponding glycones in roots were very low, especially for the scopoletin/scopolin pair, indicating that the majority of each coumarin compound is present as glucoside inside roots (Fig. 6). Although we observed a strong increase in these ratios upon Fe deficiency, reflecting higher levels of aglycones in exudates, the endogenous root levels of the respective glucosides were still higher than the respective aglycone levels in the exudates (i.e. ratios below 0.5). Only fraxetin and fraxin accumulated to almost equal levels in exudates and roots, respectively (Fig. 6). The distribution between exudates and roots dramatically changed in *pdr9-2* and *pdr8-1;pdr9-2* plants. Here, the ratios between external and internal levels of esculetin, esculin, and dihydroxyscopoletin glucoside dropped substantially (Fig. 5), as well as the ratios between aglycones in exudates and glucosides in roots for fraxetin/fraxin, esculetin/esculin, and dihydroscopoletin/dihydroxyscopoletin glucoside (Fig. 6). However, this was not observed for scopoletin and scopolin. On the other hand, disrupted ABCG36 function seemed to slightly (*P* = 0.07) affect the distribution of esculetin after Fe deficiency, as indicated by the decreased ratio in *pdr8-1* when compared to the wild-type (Fig. 6c).

## Discussion

ABC transporters are ubiquitous proteins and form a large family that greatly expanded during the evolution of plants to facilitate a wide range of physiological processes in support of their terrestrial lifestyle. Plant genomes typically encode more than 120 ABC transporters, which exceeds the number in animals by almost 3-fold, and it remains a challenging task to determine their transport properties and preferred substrates, which are often structurally and functionally unrelated^9^. In this study, we revisited the substrate specificity of two ABC transporters, ABCG36/PDR8 and ABCG37/PDR9, which specifically localize to the outermost plasma membrane of root epidermal cells and the lateral root cap^26, 27^. The study of *pdr8* and *pdr9* mutants as well as of recombinant ABCG37 protein indicated that both transporters redundantly regulate the accumulation of IBA and synthetic auxinic compounds in roots by facilitating their efflux into the rhizosphere^26, 27^. Because ABCG37 has also been implicated to promote Fe acquisition via the exudation of Fe-chelating coumarins^7, 35^, we analyzed its substrate specificity by employing a non-targeted metabolite profiling methodology of semi-polar compounds in Arabidopsis root exudates^37^, which we followed up by the targeted metabolite profiling of coumarins in root extracts and exudates.

Our non-targeted metabolite profiling study revealed that root exudate composition is clearly different between the ABC transporter mutants, *pdr8-1* and *pdr9-2*, and the wild-type (Col-0). Based on the number of differential features, both transporters seem to contribute to a similar extent to root exudation in *Arabidopsis thaliana*. However, the number of features per compound is variable and does not indicate the number of exuded metabolites. Our analysis provides only a snapshot of all possibly exuded compounds because it was restricted to semi-polar metabolites, and differences in exudation of more polar compounds, which certainly contribute to exudate profiles, are not considered as well as low abundant metabolites which escaped our detection. A limitation in sensitivity is likely the reason why reported, very low abundant substrates of ABCG36/PDR8 and ABCG37/PDR9, such as IBA or auxin^26–29^ could not be identified in this approach. We also did not detect glucosinolates or their degradation products in root exudates of any genotype. Recently, these compounds have been identified in root exudates harvested from older Arabidopsis plants^38^ using identical extraction and detection methods. It is conceivable, that glucosinolate compounds are not exuded in young seedlings or that their levels in exudates are far less abundant compared to older plants. Furthermore, glucosinolates and their degradation products were shown to accumulate upon infection with the root colonizing fungus *Piriformospora indica* ^39^. In leaves, ABCG36 was discussed to mediate glucosinolate transport and activation after pathogen attack^22^. The absence of glucosinolate signals in all investigated genotypes does not answer the question whether or not ABCG36 also mediates glucosinolate exudation by roots. It would be interesting to investigate this aspect under glucosinolate-inducing conditions. Along this line, it should generally be considered that changes in root exudation profiles of transporter mutants may only be robustly detectable after certain abiotic or biotic stimuli; otherwise, the capabilities of transporters may be underestimated.

Despite these limitations, the changes in root exudate profiles in comparison to the wild-type are quite specific for both *pdr8-1* and *pdr9-2*, indicating distinct substrate specificities of ABCG36 and ABCG37. The overlap of just 32 features between both mutants may be traced to a few compounds as one metabolite is often represented by several features; e.g., we previously showed that up to 9 features are related to dihydroxyscopoletin^37^. Overlapping substrate specificity between different ABC transporters frequently occurs and has also been reported for root exudation processes. Badri *et al*.^4^ compared the root exudate profiles of 8 different ABC transporter mutants and recorded the absence of several excreted compounds in up to 5 genotypes. Based on the number of differential features (138), the effect of the *pdr8-1;pdr9-2* double mutant on root exudation is synergistic as it clearly exceeds the sum of differential features of each single mutant (70). The strongly impaired root exudation capacity of the double mutant may be a consequence of its compromised growth, as indicated by reduced seedling, shoot, and root fresh weights as well as by shorter primary roots. However, the number of features in root exudates that are at least 1.5 fold higher compared to the blanks (*P* < 0.01) is similar for all genotypes, indicating that the double mutant is not generally compromised in root exudation because of its reduced growth. This suggests partial compensations of ABCG36 function in *pdr8-1* by ABCG37 and of ABCG37 function in *pdr9-2* by ABCG36, which are absent in the double mutant. Interestingly, we detected several features with increased intensities in root exudates of both mutant lines when compared to the wild type. These features may be related to precursors of ABCG36 or ABCG37 substrates that accumulate in *pdr8-1* or *pdr9-2* roots and are exuded by other processes. However, this scenario remains speculative until the respective compounds have been identified.

Despite the overlap and possible partial compensation of both ABCG-type transporters, we identified by our filtering procedure several features that are specifically related to ABCG36 and ABCG37 function. Among the 27 *pdr8-*specific features, one was recently identified as thymidine^38^. This compound has been detected at various levels in root exudates of different Arabidopsis accessions^40^ and its amount decreases upon root colonization with *Piriformospora indica* ^39^. The biological relevance of thymidine exudation in Arabidopsis is unclear; however, it has been suggested to function as an efficient signal to attract larvae in *Zea mays* and *Typha latifolia* ^41^. Irrespective of the unknown role of thymidine exudation in Arabidopsis, its decreased level in *pdr8-1* provides evidence that thymidine exudation is an active process and not merely a consequence of root cell death, as suggested earlier^42^.

Among the 64 *pdr9-*specific features, two were recently annotated as dihydroxyscopoletin^37^. Mass spectra^37^ (Supplementary Fig. S2), increased intensities of specific mass spectrometric signals in root exudates after Fe deficiency as well as the absence in exudates of *f6′h1-1* plants^33^ (Fig. 4), further support the identity of these features as the coumarin dihydroxyscopoletin. The observed accumulation of coumarins in roots and root exudates during Fe deficiency supports a role of this compound class for promoting Fe nutrition^7, 30–36^. However, previous reports mainly focused on coumarins with a low substitution degree, such as scopolin, scopoletin, esculetin, and esculin, whereas coumarins showing a more complex substitution pattern, such as dihydroxyscopoletin, dihydroxyscopoletin glucoside, fraxetin, fraxin, and isofraxidin have only occasionally been taken into account^7, 33, 34^. Also, tools such as the use of *f6′h1* mutants or depletion of phenolic compounds in root exudates by reverse phase columns strongly supported the importance of coumarins for Fe acquisition, but did not discriminate between individual coumarins^7, 33, 34^. Our data indicate that coumarins with more elaborate substitution patterns, especially those containing a catechol moiety, accumulate more strongly in root exudates, suggesting that these compounds might contribute considerably to Fe acquisition. Fourcroy *et al*.^7^ reported strongly impaired Fe nutrition in ABCG37 deficient *pdr9-2* and *pdr9-3* mutants, which was correlated with decreased coumarin exudation. Our results show ABCG37-dependent changes in coumarin profiles for a subset of coumarins, foremost dihydroxyscopoletin, fraxetin, esculetin, and their glucosides. Thus, our data and published work^7^ suggest that the exudation of coumarins containing a catechol moiety (esculin, fraxetin, and dihydroxyscopoletin) facilitates Fe nutrition rather than the release of monohydroxylated coumarins (scopoletin, isofraxidin). This is also supported by the work by Sisó-Terraza *et al*.^36^, where coumarins containing a catechol moiety, such as fraxetin, or coumarinolignols containing a catechol moiety, such as 5′hydroxycleomiscosin strongly accumulate upon Fe deficiency. Consistent with previous publications^33, 34, 43^, the authors also show that coumarins containing a catechol moiety chelate and mobilize Fe more efficiently.

Surprisingly, despite their comprehensive analytical approach using highly sensitive and selective methods, Sisó-Terraza *et al*.^36^ did not detect dihydroxyscopoletin. Exposure of roots to light in our hydroponic system might be a reason. However, light only poorly penetrates the outer wall of our hydroponic containers (Supplementary Fig. S3), and it remains to be investigated, whether the low light intensities reaching the roots are actually responsible for the generation of dihydroxyscopoletin. Also, oxygen availability could account for differences in the detection of compounds. This seems to be unlikely, however, since aerated nutrient solutions, which were weekly changed, were used in both systems. Whether accumulation of dihydroxyscopoletin depends on the age of plants remains to be investigated as well. In our study, plants and exudates were harvested 11 days after germination, in contrast to 35 days^36^. If this was the case, it would be interesting to elucidate whether the lack of dihydroxyscopoletin at later stages of plant growth might be due to decreased biosynthesis or increased metabolization.

There is a strong prevalence of coumarin glucosides in roots and of aglycones in exudates^33^ (Figs 3–5), suggesting that coumarins are transported in their free form. However, the presence of glucosides in exudates and the occurrence of coumarin glucoside specific hydrolyzing ß-glucosidases with predicted apoplastic localization^44, 45^ indicate glucoside transport and subsequent hydrolysis. The decreased and increased levels of esculin and dihydroxyscopoletin glucoside in *pdr9-2* root exudates and roots, respectively, also imply that coumarin glucosides might constitute the transported compounds. Whether this is also true for fraxin cannot be concluded from our experiments because it escaped detection in root exudates. The *bglu42* mutant, containing a T-DNA insertion in a gene coding for a root-epidermis localized ß-glucosidases is impaired in the Fe deficiency-induced secretion of fluorescent compounds to the rhizosphere^46^. This result suggests that glucoside hydrolysis precedes exudation. However, the chemical identities of the fluorescent compounds were not investigated in this study, so that it still remains an open question, whether or not BGLU42 mediated hydrolysis is a prerequisite of coumarin transport, and if so, whether there is a specificity with respect to the substitution patterns of the coumarin core structure. Whatever form is transported, our results clearly show selectivity of ABCG37 *in vivo* with respect to the substitution pattern of the coumarin core structure. As such, ABCG37 preferentially mediates the transport of coumarins with a catechol moiety, such as dihydroxyscopoletin, esculetin, and fraxetin or their glucosides, whereas coumarins with one hydroxyl group, such as scopoletin or its glucoside and isofraxidin are not accepted as substrate. However, it should be considered that additional factors, such as coumarin metabolism or presence and absence of competing compounds, might have an impact on the observed coumarin profiles in the two ABCG transporter mutants. For example, Fourcoy *et al*.^7^ observed strongly reduced levels also of isofraxidin and scopoletin in root exudates of *pdr9-2* plants. Compared to our experimental setup, their plants were 14 days older with a more developed root system at the onset of Fe deficiency. Also, Fe deficiency induced scopoletin exudation seemed to be much stronger compared to our results. Thus, it is possible that the different observations regarding *pdr9-2* dependent coumarin profiles are due to additional factors, which do not affect ABCG37 transport selectivity per se. Therefore, considering these inevitable drawbacks, the transport specificity observed in *in vivo* experiments using transporter mutants might differ from the actual selectivity of the transporter proteins for distinct compounds, which ultimately can only be assessed using *in vitro* transport assays.

## Methods

### Reagents and Standards

All buffer and media components and solvents were of reagent or HPLC grade and obtained from Sigma-Aldrich (St. Louis, MO, USA), J.T. Baker (Deventer, The Netherlands), and Roth (Karlsruhe, Germany). Fraxetin, fraxin, and isofraxidin were purchased from PhytoLab (Vestenbergsgreuth, Germany) and Sigma-Aldrich.

### Plant Lines and Growth Media

*Arabidopsis thaliana* accession Columbia (Col-0) and the Col-0 mutant lines *pdr9-2* (At3g53480; SALK_050885), *pen3-4*, here referred to as *pdr8-1* (At1g59870; SALK_000578), the *pdr8-1;pdr9-2* double mutant as well as *f6′h1-1* (At3g13610; SALK_132418 C) have been described previously^20, 27, 28, 33, 37^. Seeds were surface sterilized with chlorine gas and placed individually into the wells of a 96-well PCR plate filled with agar according to the recently developed sterile hydroponic system^37^(Supplementary Fig. S3). The aerated growth medium contained 5 mM KNO_3_, 2.5 mM KH_2_PO_4_, 2 mM MgSO_4_, 2 mM Ca(NO_3_)_2_, 50 µM Fe^3+^-EDTA, 70 µM H_3_BO_3_, 14 µM MnCl_2_, 0.5 µM CuSO_4_, 1 µM ZnSO_4_, 0.2 µM Na_2_MoO_4_, 10 µM CoCl_2_ and 5 g l^−1^ sucrose, pH 5.6. Agar (Duchefa, Haarlem, The Netherlands) was routinely purified^47^ and added at a concentration of 0.75% (w/v). After two days of stratification at 4 °C in the dark, the hydroponic systems were placed on a rotary shaker (80 rpm) in a growth chamber at 22 °C under illumination for 16 h daily (170 µmol s^−1^ m^−2^; Osram LumiluxDeLuxe Cool Daylight L58W/965, Osram, Augsburg, Germany). After 4 days, the PCR plates containing the germinated seedlings were transferred to fresh medium without sucrose. For Fe deficiency treatment, Fe^3+^-EDTA was omitted from the fresh medium. After additional 7 days of growth, plants and exudates were harvested.

### Non-Targeted Metabolite Profiling of Semi-Polar Compounds

One experiment consisted of five replicates per genotype, in which one replicate represented the exudates of 8 plants, and 3 experiments were performed in a 3 month interval. In total, the number of replicates was 13 for wild-type and *pdr9-2*, 15 for *pdr8-1*, and 8 for *pdr8-1;pdr9-2*. Additionally, we collected the media of 15 blank samples, i.e. positions in the 96 well hydroponic system that did not receive seeds. Per replicate, the exudates from 8 roots were combined, spiked with 100 nmol 2,4 dichlorophenoxy acetic acid (Duchefa) and all replicates were processed simultaneously as described^37^. Non-targeted metabolite profiling was performed according to Strehmel *et al*.^38^. Only datasets passing the quality check (retention time shift < 5 s and intensity deviation < 30% of the internal standard, 2,4 dichlorophenoxy acetic acid) were considered for further analysis. Replicates for each genotype were grouped, and data were processed using the R package XCMS^48^ with an intensity threshold of 1,000 and a minfrac value of 1. Features (*m/z* retention time pair) were regarded as different between genotypes if the median intensities differed by more than 1.5 fold at *P* < 0.01 (Student’s *t*-test, two-tailed, equal variance).

### Targeted Coumarin Profiling

Extraction, measurement, and quantification of coumarins were performed as described^37^ using 4-methyl-umbelliferon as standard. Quantifier and qualifier transition as well as compound specific parameters for fraxin, fraxetin, and isofraxidin are listed in Supplementary Table S2.

## Electronic supplementary material

Supplementary info

Dataset
