# Supplementary material for: Arabidopsis Transporter ABCG37/PDR9 contributes primarily highly oxygenated Coumarins to Root Exudation
Source: Sci Rep. 2017 Jun 16;7:3704. doi: 10.1038/s41598-017-03250-6 (PMC5473935; doi:10.1038/s41598-017-03250-6)
Supplement: Supplementary file 1 — Supplementary info [file 41598_2017_3250_MOESM1_ESM.pdf]

Supplementary Information

Arabidopsis Transporter ABCG37/PDR9 contributes primarily highly oxygenated Coumarins to Root Exudation

Jörg Ziegler[1,*], Stephan Schmidt[2], Nadine Strehmel[2,], Dierk Scheel[2], and Steffen Abel[1,3,4]

[1] Department of Molecular Signal Processing, Leibniz Institute of Plant Biochemistry, D-06120 Halle (Saale), Germany

[2] Department of Stress and Developmental Biology, Leibniz Institute of Plant Biochemistry, D-06120 Halle (Saale), Germany

[3] Institute of Biochemistry and Biotechnology, Martin Luther University Halle Wittenberg, D-06120 Halle (Saale), Germany

[4] Department of Plant Sciences, University of California-Davis, Davis, CA 95616, USA

Supplementary Figure S1

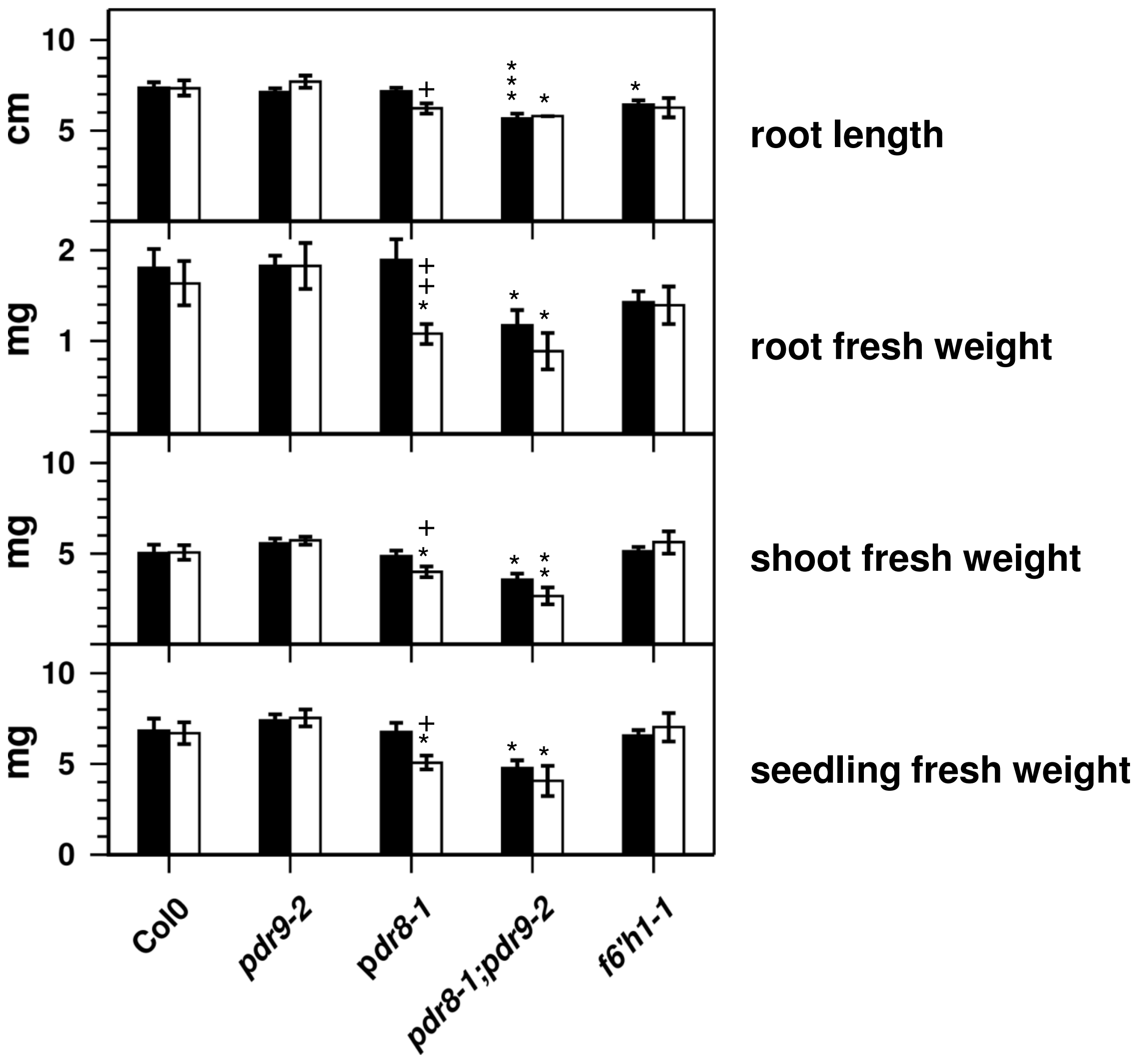

Supplementary Figure S1. seedling, shoot, root fresh weights and root lengths of 11 days old seedlings, either grown in full medium (+Fe) (solid bars), or after transfer to –Fe medium (open bars) at day 4. Error bars denote SEM (*n*=8 for fresh weights, *n*=16 for root lengths; for *pdr8-1;pdr9-2* root length –Fe: *n*=5. Significance analysis was performed by Student's *t*-test (two tailed, equal variance). *: *P*≤0.05; **: *P*≤0.01 ***: *P*≤0.001 for comparison between mutants and Col0; + : *P*≤0.05; ++ : *P*≤0.01 between + and – Fe treatment

Supplementary Figure S2

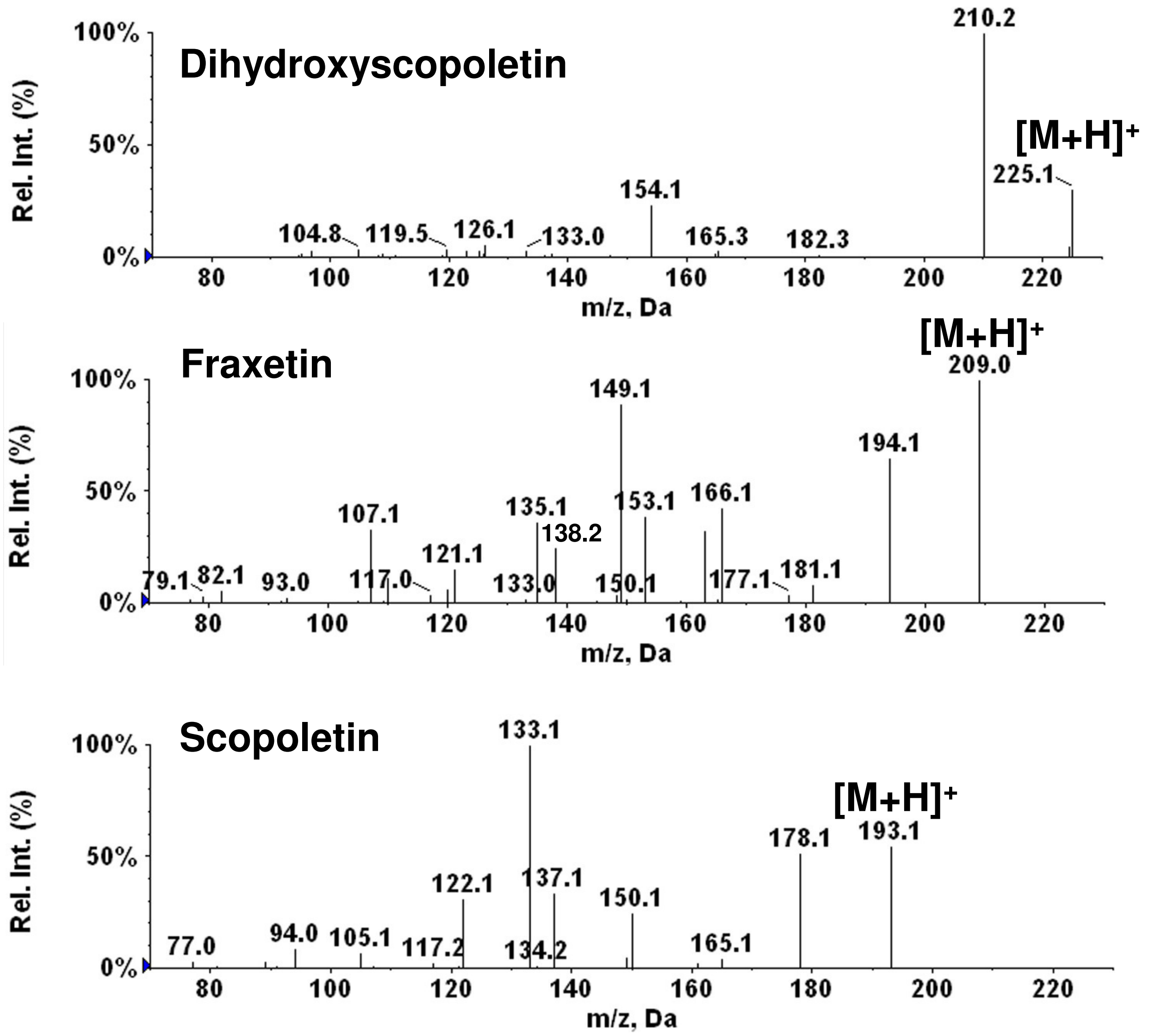

Supplementary Figure S2. MS^2 spectra of the authenticated standards fraxetin, scopoletin, and of the compound tentatively assigned as dihydroxyscopoletin. The mass spectrum of the tentative dihydroxyscopoletin was recorded at a retention time of 2.5 min after the injection of an exudate sample showing a strongly increased signal for *m/z* 225 Da after seedlings have been exposed to Fe-deficiency. All spectra were recorded using a collision energy of 30V in the positive ionization mode.

Supplementary Fig. S3: The hydroponic system used in this study

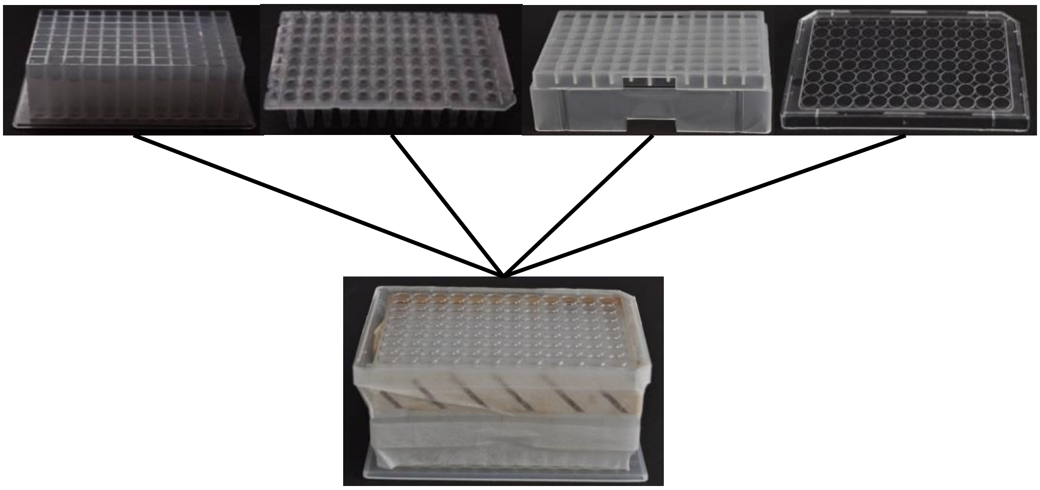

Supplementary Fig. S3: Assembly of the hydroponic system.

Supplementary Table S1:

Number of datasets for each genotype

experiment	ionization	Col0	*abcg36 (pdr8-1)*	*abcg37 (pdr9-2)*	*abcg36;abcg37 (pdr8-1;pdr9-2)*	blanks
1	positive	5	5	4	1	5
	negative	5	5	4	1	5
2	positive	4	5	4	1	5
	negative	4	5	4	1	5
3	positive	4	5	5	4	5
	negative	4	5	5	4	5
Σ	positive	13	15	13	6	15
	negative	13	15	13	6	15

pdr8-1: SALK_000578[1]

pdr9-2: SALK_050885[2]

pdr8-1;pdr9-2[3]

1 Kobae, Y. *et al.* Loss of AtPDR8, a plasma membrane ABC transporter of *Arabidopsis thaliana*, causes hypersensitive cell death upon pathogen infection. *Plant Cell Physiol.* **47**, 309-318 (2006).

2 Ito, H. & Gray, W. M. A gain-of-function mutation in the *Arabidopsis* pleiotropic drug resistance transporter PDR9 confers resistance to auxinic herbicides. *Plant Physiol.* **142**, 63-74 (2006).

3 Růžička, K. *et al. Arabidopsis* PIS1 encodes the ABCG37 transporter of auxinic compounds including the auxin precursor indole-3-butyric acid. *Proc. Natl. Acad. Sci. USA* **107**, 10749-10753 (2010).

Supplementary Table S2 MS parameters for MRM-transitions

Coumarins

	MRM transitions	Retention time, min	Declustering potential (DP), V	Entrance potential (EP), V	Cell entrance potential (CEP), V	Collision potential (CE), V	Cell exit potential (CEX), V
4-Methyl umbelliferon	**177→103**	3.8	51	3.5	10	33	4
	177→105		51	3.5	10	27	4
Esculetin	**179→123**	2.5	51	10.5	14	31	4
	179→133		51	10.5	14	27	4
Esculin	**341→179**	2.0	46	4.5	22	23	4
	341→123		46	4.5	22	59	4
Scopoletin	**193→133**	3.2	51	3.5	12	29	4
	193→178		51	3.5	12	29	4
Scopolin	**355→193**	2.4	26	4.5	24	19	4
	355→133		26	4.5	24	55	4
Dihydroxy scopoletin	**225→210**	2.5	25	9	15	30	4
	225→136		25	9	15	30	4
Dihydroxy scopoletin-glucoside	**387→225**	2.3	30	3	20	20	4
	387→210		30	3	20	45	4
Fraxin	**371→209**	2.5	26	2.5	18	19	4
	371→194		26	2.5	18	49	4
Fraxetin	**209→149**	2.8	51	4.5	8	29	4
	209→194		51	4.5	8	27	4
Isofraxidin	**223→162**	3.4	51	4.5	14	33	4
	223→190		51	4.5	14	29	4

Quantifier and qualifier transitions are indicated in bold and italics,
